# A flexible organic mechanoluminophore device

**DOI:** 10.1038/s41467-023-36916-z

**Published:** 2023-03-06

**Authors:** Qingyang Zhang, Mengxin Xu, Liming Zhou, Shihao Liu, Wei Wang, Letian Zhang, Wenfa Xie, Cunjiang Yu

**Affiliations:** 1 State Key Laboratory of Integrated Optoelectronics, College of Electronic Science and Engineering, Jilin University, Changchun, 130012 China; 2 School of Mechanical and Aerospace Engineering, Jilin University, Changchun, 130025 China; 3 Department of Engineering Science and Mechanics, Pennsylvania State University, University Park, PA 16802 USA; 4 Department of Biomedical Engineering, Pennsylvania State University, University Park, PA 16802 USA; 5 Department of Material Science and Engineering, Materials Research Institute, Pennsylvania State University, University Park, PA 16802 USA

**Keywords:** Electrical and electronic engineering, Optoelectronic devices and components

## Abstract

A flexible mechanoluminophore device that is capable of converting mechanical energy into visualizable patterns through light-emission holds great promise in many applications, such as human-machine interfaces, Internet of Things, wearables, etc. However, the development has been very nascent, and more importantly, existing mechanoluminophore materials or devices emit light that cannot be discernible under ambient light, in particular with slight applied force or deformation. Here we report the development of a low-cost flexible organic mechanoluminophore device, which is constructed based on the multi-layered integration of a high-efficiency, high-contrast top-emitting organic light-emitting device and a piezoelectric generator on a thin polymer substrate. The device is rationalized based on a high-performance top-emitting organic light-emitting device design and maximized piezoelectric generator output through a bending stress optimization and have demonstrated that it is discernible under an ambient illumination as high as 3000 lux. A flexible multifunctional anti-counterfeiting device is further developed by integrating patterned electro-responsive and photo-responsive organic emitters onto the flexible organic mechanoluminophore device, capable of converting mechanical, electrical, and/or optical inputs into light emission and patterned displays.

## Abstract (web summary)

It has been challenging to achieve discernible mechanoluminophore devices under ambient light. Here, authors integrate top-emitting organic light-emitting device and piezoelectric generator on thin polymer substrate for the realization of flexible devices under an ambient illuminance of 3000 lux.

## Introduction

A flexible mechanoluminophore device that displays desired patterns by light emission based upon mechanical stimulations holds great promise in a wide range of applications, ranging from human-machine interfaces^1^, to smart textile^2^, to health monitoring^3^, to wearables^4^, to anti-counterfeiting^5^, and to self-powered displays^6^. Primarily motivated to visualize the mechanical stress on materials and structures, there has been a growing interest in developing mechanoluminescence (ML) materials^7–10^. Although there are some advances on mechanoluminophore materials and devices^7–18^, a soft, flexible counterpart has been very nascent, and more importantly, it needs to be discernible under ambient light. Existing mechanoluminophore materials and devices are mainly categorized into three types based on different working mechanisms. The ML materials are primarily based on piezophotonic effect directly converting the external mechanical energy into light emission without the assistance of electron or photon excitation^4,5,11^. The second is triboelectrification-induced electroluminescence (TIEL), which owes to the coupling effect between triboelectrification and electroluminescence (EL)^12–16^. The triboelectric charges yielded from contacting and/or sliding behavior at the interfaces of the TIEL materials generate a strong electric field in the emitting material that leads to electron transitions into luminescence center and thus generates EL^19^. The third is the piezoelectric-induced electroluminescence (PIEL), where a piezoelectric generator converts mechanical energy generated from mechanical motions such as compressionn and/or vibration, into electric energy thus powering light emitting devices^17,18^. However, a critical challenge in these mechanoluminophore devices is their low light intensity which makes them indiscernible under ambient light^20–25^. Moreover, to optimize the structure and material design of mechanoluminophore devices, a standard characterization method is necessary to be established for extracting useful information from different researches^3,19,26,27^.

Here we report the development of a low-cost flexible organic mechanoluminophore device, which is constructed based on the integration of a high-efficiency, high-contrast top-emitting organic light-emitting device (TEOLED) and a piezoelectric generator (PG) of polyvinylidene difluoride (PVDF) thin film on a thin poly (ethylene-naphthalate) (PEN) substrate. The thin PEN substrate not only serves as a supporting substrate, but confers favorable stress states in the PG to generate a millisecond current pulse with an amplitude of 0.64 mA cm^−2^ to activate the OLED. By introducing a high-absorption 5, 6, 11, 12-tetraphenylnaphthacene (rubrene) capping layer to the TEOLED and precisely adjusting the microcavity effect, high-brightness and high-contrast TEOLED is achieved to ensure the discernibility even under high ambient illumination (3000 lux) comparable to a cloudy environment. Distinguished from the existing ML materials and devices^3,7–19,24–27^, our organic mechanoluminophore device provides three modes of operation: mechanoluminescence, electroluminescence and photoluminescence (PL). Through rational masking and structure design, PIEL and PL in the same area of the device can even display different patterns or color. All together, a flexible multifunctional anti-counterfeiting device is developed by integrating patterned electro-responsive and photo-responsive organic emitters into the flexible organic mechanoluminophore device, capable of converting mechanical, electrical, and/or optical inputs into light emission and patterned displays.

## Results

### Construction of a flexible organic mechanoluminophore device

**Fig. 1:**
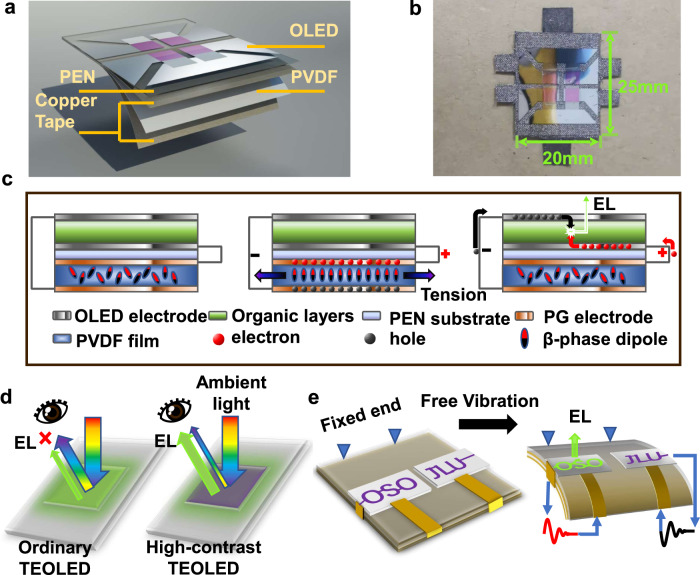
The flexible mechanoluminophore device. **a** The schematic illustration in an explored view of the device’s layered structure; **b** the photo and dimensions of the device. **c** Three states of the device’s working principle. Left: non-operating state, Middle: piezoelectric polarization state under the action of internal stress in the piezoelectric generator (PG), and Right: charge transfer and injection into the light-emitting device to cause light emission (EL, electroluminescence). **d** Schematic diagram of the EL (green arrows) visibility of ordinary and high-contrast TEOLEDs to human eyes under ambient light noise (rainbow arrows). **e** Schematic diagram of the generation process of patterned light-pulse anti-counterfeiting signal. Patterns in purple refer to off state while green pattern refers to EL generation. Red and black waveform indicate two directions of the power supply.

We developed a flexible organic mechanoluminophore device by directly integrating a piezoelectric generator (PG) with a TEOLED. It is noted that a PG has been utilized to power commercially available inorganic LEDs as reported previously^28^, which further proves the feasibility of integrating these aforementioned two components towards a flexible organic mechanoluminophore device upon rational designs. Figure 1a, b shows the schematic illustration and an optical image of the flexible organic mechanoluminophore device, respectively. The device has an overall size of 25 mm × 20 mm and the four TEOLED units have an area of 10 mm^2 ^each. The PG is based on a Cu tape/Al/PVDF/Al/Cu tape/PEN multilayered structure, working under the *d*_31_ mode. The PEN flexible substrate seats in between the PG and the TEOLED. Aluminized electrodes on both sides of the PVDF film were used to provide good electrical contact thus to reduce the dissipation of generated power. Since the PVDF film has a high coefficient of thermal expansion (CTE)^29^, directly evaporating thin films of TEOLEDs on its top would result in wrinkle structures once they cool down to room temperature, which would significantly impact the TEOLED performance since high substrate uniformity is a critical requirement. To avoid the formation of wrinkle structures and meet the need for TEOLED performance, a 125 μm thick flexible PEN substrate with low surface roughness (root mean square 1–2 nm)^30^ and high thermal stable temperature (>140 °C), low CTE (~10.2 ppm K^−1^)^31^ is therefore employed as the substrate. The PVDF and TEOLEDs are connected by copper fabric tapes.

As shown in Fig. 1c (left image), at the initial state, the β-phase dipoles of the PVDF layer are in an electrically neutral arrangement, and no piezoelectric induced charges are generated. Therefore, no current flows in the circuit, thus the OLED is at an off state. When a transverse stress acts on the PVDF, the dipoles are rearranged, leading to positive and negative charges induced on both electrodes of the PG (Fig. 1c, middle)^32–34^. The induced charges generate a potential difference between the two electrodes of the TEOLED^35^, which in turn facilitates the charge injections to the cathode and anode. As shown in Fig. 1c (right image), under the induced electrical potential, the injected charges overcome the potential barrier to form exciton pairs, which are recombined and quickly consumed in the organic light-emitting layer. Once the deformation is released, the dipoles in piezoelectric layer returns to the initial state. The response speed of stress-charge-light conversion in this flexible mechanoluminophore device is mainly governed by the average carrier lifetime of the TEOLED.

We introduced free vibration to the one-end-fixed piezoelectric film to generate the induced charge, which is expected to create higher energy density through low frequency and small amplitude external force inputs like finger flicking and bending in real applications. Without an extra power storage unit, a rectifying circuit or an amplifier circuit, the free vibration results in an underdamped alternate piezoelectric voltage/current. It is noted that the TEOLED unit emitted light pulses rather than constant light emission, similar to the reported AC-driven ML devices^36–38^. In order to ensure that the flexible mechanoluminophore device can be discernible under ambient light, a high-absorption capping layer is proposed. In Fig. 1d, the capping layer extracts more light emission meanwhile reflects less ambient light, which promotes the contrast and EL efficiency. Under opposite initial deflections, the freely vibrating PG generates an opposite underdamped alternating current (AC) voltage, which in turn lits up the TEOLED unit with opposite electric connections, as shown in Fig. 1e.

**Fig. 2:**
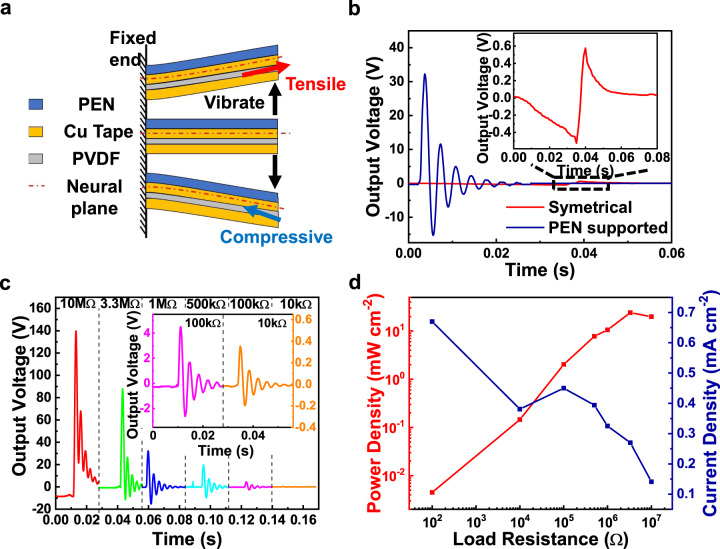
Performance optimization and characterization of PVDF piezoelectric generator. **a** Schematic diagram of the neutral plane of a multilayer system and its effect on how internal stress react to free vibration behavior. **b** Comparison of piezoelectric output with and without (symmetrical structure) PEN support layer. **c** Output voltage-time curve under different loads. The inset is an enlarged view of the voltage curve for 100 kΩ and 10 kΩ loads. **d** The curve of output power and current density-load resistance. Source data are provided in a Source Data file.

We measured the thickness and bending elastic modulus of each constituent layer (Supplementary Table 1) and calculated the position of the neutral mechanical plane (Supplementary Fig. 1a, b and Supplementary Note 1). It can be seen from Fig. 2a that the neutral mechanical plane is located near the PEN substrate, so that the stress within PVDF layer will be fully compressive or tensile during vibration, avoiding charge cancellation and thus enhancing the output voltage, as illustrated in Fig. 2b. The <3, 1> direction is adopted as the PG operating orientation, under which mode the device can produce three times higher output voltage than that of the <3, 2> direction^39^, as shown in Supplementary Fig. 1c.

According to the Supplementary Note 1 and Supplementary Fig. 1d, as the length *l* of the PG increases from 4 to 20 mm, the free vibration frequency decreases from 3700 to 197 Hz, while the highest output voltage is obtained when the PG length *l* = 16 mm, with a PG output frequency of ~280 Hz. These results match with the finite element simulations results (Supplementary Fig. 2b). Since the overall thickness of the OLED device is much lower than that of the PG, the integrated device can be modeled as a simple laminated piezoelectric film in simulation, despite the influence of the OLED. The finite element results, equivalent circuit, and equations applied in calculating the voltage output with a 1 MΩ load are shown in Supplementary Fig. 2a, b and Supplementary Note 2, respectively.

Under the load of 1 MΩ, the output voltage of the PG reaches 33 V when *l* = 16 mm. As shown in Fig. 2c, the output voltage monotonically increases as the load resistance is higher. Under the load of 10 MΩ, the proposed PG can be regarded as an open-circuit, and a voltage up to 140 V is obtained. Since the load is essentially in parallel with large PG body resistance and PG capacitor, the output current will slightly decrease when the load increases. Under the load of 100 Ω, a peak current density of 0.67 mA cm^−2^ is obtained as shown in Fig. 2d, which is sufficient to activate a green OLED (~10 mm^2^, 512 nm, brightness >1 cd cm^−2^). It is worth mentioning that other flexible piezoelectric materials such as PVDF/ZnO composite nanofibers^40^, P(VDF-TrFE) polymer^41^, and biomaterials (e.g., fish gelatin^42^, cellulose-based materials^43^, etc.) might also be appropriate as long as their stability, piezoelectric coefficient, and mechanical strength is suitable^44,45^.

### Discernibility analysis and TEOLED optimization

The discernibility of a flexible mechanoluminophore device under an ambient environment is challenging. Since the PG generates an underdamped sine wave with a frequency of ~280 Hz when *l* = 16 mm, the OLED emits a light pulse only at the first positive voltage peak. The large duty cycle between repeated light pulses will make the emission of the OLED greatly different from that of traditional direct current (DC)-driven OLEDs, as can be seen in Supplementary Fig. 3b. More importantly, critical challenges lie in the lack of a systematic analysis of brightness and discernibility of these AC-driven flexible mechanoluminophore device. Here, we have established a quantitative analysis method of the discernibility of the flexible mechanoluminophore device, which includes the equivalent brightness calculation of light pulse and the just-noticeable-ambient-illuminance (JNAI) considering ambient light reflection on device.

In the AC mode, the EL intensity of OLED is a combination of periodic pulses, with a shrinking duty cycle as the power source frequency increases, as shown in Supplementary Fig. 3a. When the frequency gradually approaches the relaxation time of OLED charge injection and light emission, the carriers that have not completed the light-emitting process in the last cycle will be neutralized by the reverse carriers, resulting in a decrease in the overall EL intensity^46,47^. Supplementary Figure 3b indicates that the EL intensities of OLEDs driven by AC sinusoidal voltage and even square wave voltage are lower than those of the DC at the same voltage amplitude. The equivalent brightness of AC OLEDs is obtained by the Talbot-Plateau law^48^: ${I}_{{{{{{\rm{E}}}}}}}{|}_{f}=\frac{1}{T}{\int }_{0}^{T}{I(t){{{{{\rm{|}}}}}}}_{f}{{{{{{\rm{d}}}}}}t}$*,* where ${I(t){{{{{\rm{|}}}}}}}_{f}$ is the OLED instantaneous EL intensity as a function of time at frequency $f$^49^. Compared to the DC mode, high duty cycle makes the effective brightness of the AC mode much smaller (Supplementary Fig. 3c).

With nearly infinite duty cycles, the EL intensity of the flexible mechanoluminophore device should be measured using the physiological equivalent light intensity of a single light pulse. From the Schmidt-Clawson law, the physiological equivalent light intensity ${I}_{{{{{{\rm{E}}}}}}}$ of a light pulse is given by ${I}_{{{{{{\rm{E}}}}}}}=J/(c+J/{I}_{0})$, where $J={\int }_{{t}_{1}}^{{t}_{2}}I\left(t\right){{{{{{\rm{d}}}}}}t}$ is the integrated instantaneous light intensity within the given duration of a light pulse. $c$ is the visual time constant, which is 0.2 s in dark environment and 0.1 s in bright environment. ${I}_{0}$ is the peak EL intensity, and it is lower than that of the DC mode^49^. These, as a result, make the equivalent light intensity of light pulse at the same driving voltage much smaller than that of the AC mode, and two orders of magnitude smaller than that of the DC mode (Supplementary Fig. 3c).

Many existing flexible mechanoluminophore devices can work successfully in darkroom environment, but this is not enough for all-weather applications. In addition to the brightness factor of the device, external light interference in bright environments needs to be considered. *R*_L_, Luminous reflectance of a device is defined as ${R}_{{{{{{\rm{L}}}}}}}=\frac{{\int }_{380}^{780}\,V(\lambda )S(\lambda )R(\lambda ){{{{{\rm{d}}}}}}\lambda }{{\int }_{380}^{780}\,V(\lambda )S(\lambda ){{{{{\rm{d}}}}}}\lambda }$, where the $V\left(\lambda \right)$ is the normalized photopic response of human eye, $S(\lambda )$ is the spectral power distribution of the ambient light source, and $R(\lambda )$ is the optical reflection of the device^50^. The simulated optical reflection of TEOLEDs with different cavity lengths is shown in Supplementary Fig. 4a. Two kinds of ambient light sources, including a white LED and a D65 source simulating sunlight scattering into the interior, are shown in Supplementary Fig. 4b.

Since the luminescence of TEOLEDs under bright environment observed by human eyes is a superposition of the device EL and *R*_L_, the Weber–Fechner’s law, $\frac{\triangle L}{L{R}_{{{{{{\rm{L}}}}}}}}=k({{{{{\rm{const}}}}}})$, is introduced to analyze the discernibility limits^51^. $\triangle L={I}_{{{{{{\rm{E}}}}}}}$ represents the brightness fluctuation (or just-noticeable-brightness-difference) in the ambient illuminance *L* (or JNAI). The ratio of $\triangle L$ to $L{R}_{{{{{{\rm{L}}}}}}}$ (the ambient light reflected on the target surface and entering human eyes) is a constant called Weber’s fraction *k* (0.01 in general). It indicates that reducing the luminous reflectance or increasing the brightness of TEOLEDs can improve JNAI. Therefore, a high-contrast and high-efficiency TEOLED is necessary.

**Fig. 3:**
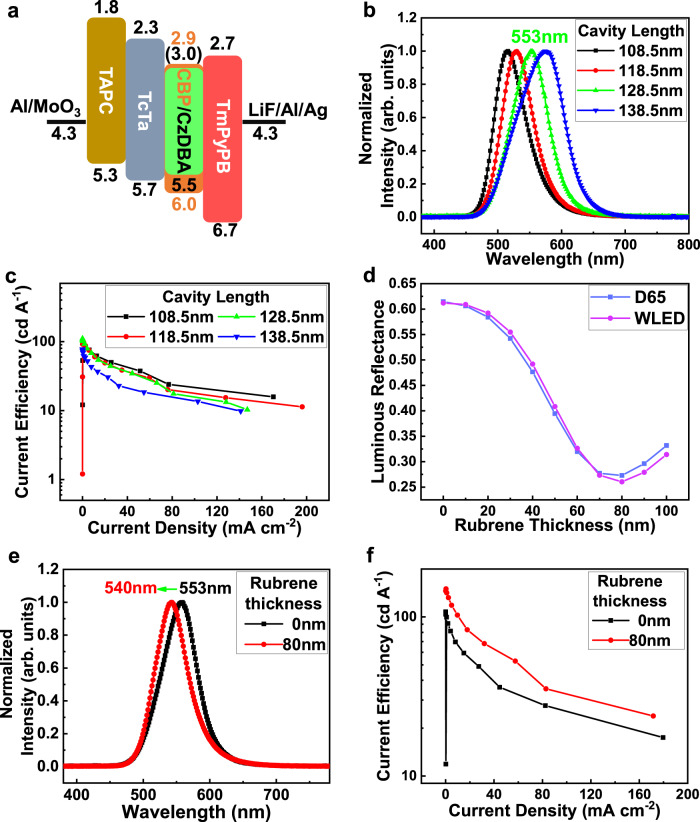
TEOLED optimization through microcavity and light extraction modulating. **a** The energy level alignment. **b** The normalized EL spectra of TEOLEDs with different cavity lengths. **c** Current efficiency-current density curves of TEOLEDs with different cavity lengths. **d** Theoretical calculation of the luminous reflectance of TEOLED with 128.5 nm cavity length as a function of the thickness of rubrene. The blue and violet symbol-lines refers to the conditions of D65 (sunlight) and WLED (indoor lighting) ambient light sources, respectively. **e** Normalized EL spectra and **f** current efficiency-current density curves of TEOLEDs with or without rubrene. Source data are provided in a Source Data file.

Green thermally activated delayed fluorescence material 5,10-bis(4-(9H-carbazole-9-yl)-2,6-dimethylphenyl)−5,10-dihydroboranthrene (CzDBA) was used as the light emitter, which has been proven to exhibit high internal quantum efficiency and relatively short exciton lifetime^52,53^. By modulating the microcavity length of the TEOLED to 128.5 nm, the resonance wavelength was adjusted to 553 nm, close to the wavelength of the maximum photopic response (555 nm)^54^. The highest current efficiency is also obtained. Besides, the thickness modulation of the organic functional layer results in a reduced carrier accumulation, thus enhancing the OLED performance^55^. Figure 3a shows the energy level structures. Figure 3b presents the normalized EL spectra of the TEOLED with different cavity lengths. It can be seen from Supplementary Fig. 5a that a turn-on voltage as low as ~3 V and a current density <1 μA cm^−2^ are obtained as the cavity length gradually increases. A current efficiency of up to 110 cd A^−1^ is obtained at a cavity length of 128.5 nm, as shown in Fig. 3c. When the optimized TEOLED is connected to the PG, green light pulses can be successfully observed, as shown in Supplementary Movie 1.

Adding a capping layer on the top electrode is an effective method to improve the efficiency^56^ or contrast^57^ of TEOLEDs. In this report, amorphous rubrene film with high absorption at 450–540 nm is used as the capping layer to simultaneously satisfy the low luminous reflectance and high efficiency of TEOLEDs. The simulated optical reflection of the TEOLEDs with different thicknesses of rubrene is shown in Supplementary Fig. 4c. Compared with Supplementary Fig. 4a, a significant reduction of reflectance in the range of the OLED EL peak (500–600 nm) is observed. Figure 3d shows the luminous reflectance of the TEOLED with different thicknesses of rubrene at a vertical viewing angle. It can be seen that a low luminous reflectance of 0.27 is obtained in the device with an 80 nm rubrene capping layer. This can also be verified from the photos of the devices shown in Supplementary Fig. 4d. Simultaneously, the front current efficiency up to 150 cd A^−1^ is obtained in the TEOLED with an 80 nm rubrene capping layer, as shown in Fig. 3f. Compared with bared devices, a narrower spectrum, and 13 nm blue-shift are observed, as shown in Fig. 3e, which indicates that the capped device has a stronger microcavity effect and improved current efficiency.

### Performances of the flexible organic mechanoluminophore device

**Fig. 4:**
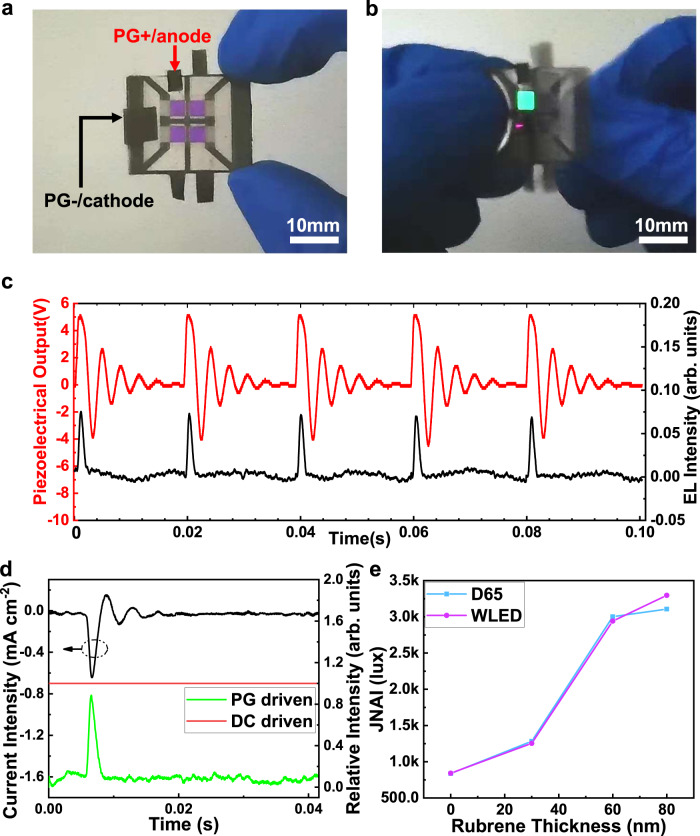
Characteristics of flexible mechanoluminophore device. **a** Schematic diagram of PG electrode connection to TEOLED, where PG+ and PG− refer to the PG positive and negative electrodes, respectively. **b** A picture of the TEOLED light pulse generation when PG is actuated. **c** Piezoelectric output voltage and normalized photoelectric signal for multiple cycles. **d** Upper black curve: The current density of an TEOLED driven by PG. Middle red and lower green curves: the relative EL intensity of constant light (DC driven) and light pulse against time. **e** JNAI of the mechanoluminophore devices with different thicknesses of rubrene under D65 and white LED ambient light sources. Source data are provided in a Source Data file.

By assembling the PG with the optimized TEOLEDs evaporated on a PEN substrate, the flexible mechanoluminophore device is obtained, as shown in Fig. 4a. Actuating the device in free vibration mode by finger flicking, the TEOLEDs emit distinct green light pulses that can be observed by naked eyes, as shown in Fig. 4b. Figure 4c shows the light pulse intensity and corresponding voltage of TEOLEDs. Due to the existence of electron blocking layer and the mismatch of hole and electron transporting properties in the organic layer, there will be a large accumulation of holes at the first positive voltage peak^58^. The accumulated holes create a reverse electric field, so that the positive peak is attenuated compared to the purely resistive load case. At the same time, due to the immediate arrival of negative AC peak, the accumulated charge is quickly neutralized, resulting in a lower peak luminous intensity than that of DC TEOLEDs, as shown in Fig. 4d. The PG peak voltage reaches 5 V while the peak current intensity reaches 0.64 mA cm^−2^ in Fig. 4c, d, respectively. The operating window for the TEOLED (that is, the time span where an applied voltage is larger than TEOLED turn-on voltage) reaches millisecond level, so TEOLED units generate light pulses at the first positive peak. Although the amplitude of the first negative peak is higher than the turn-on voltage, the TEOLED cannot be activated as it is in a reverse-biased cut-off state. The amplitude of the second positive peak is smaller than the turn-on voltage due to the damping mechanism of the PG, so the TEOLED still cannot be activated. During a complete free vibration process, the flexible organic mechanoluminophore device emits only one light pulse, which greatly improves the recovery speed of the electro-optical response.

As shown in Supplementary Fig. 5b and Supplementary Note 3, the *I*_E_ of the integrated flexible mechanoluminophore device is calculated by an equivalent resistance method, for quantitatively characterizing the performance in a bright environment. Thus, the ${I}_{{{{{{\rm{E}}}}}}}$ of 11.5 cd m^−2^ is obtained, which also equals to ∆*L* in the Weber–Fechner’s law. Then, the JNAI of the flexible mechanoluminophore device with different thicknesses of rubrene can be calculated and is shown in Fig. 4e. A JNAI as high as ~3000 lux is obtained when the rubrene thickness reaches 80 nm. In general, the ambient illuminance of a bright indoor environment is about 400 lux, which is much smaller than the calculated results. Therefore, based on experiments and theoretical calculations, we have proved that the proposed device is able to work in a bright environment, superior to known flexible mechanoluminophore devices. Additionally, a cyclic bending test has been carried out to benchmark the mechanical durability of the flexible organic mechanoluminophore device. As shown in Supplementary Fig. 6 and Supplementary Movie 2, a device was bent over 1300 times under ambient conditions, and its operation was intact. Further encapsulation could additionally improve the stability of OLED devices against temperature, humidity, and other environmental influences^59^.

### Anti-counterfeiting device

**Fig. 5:**
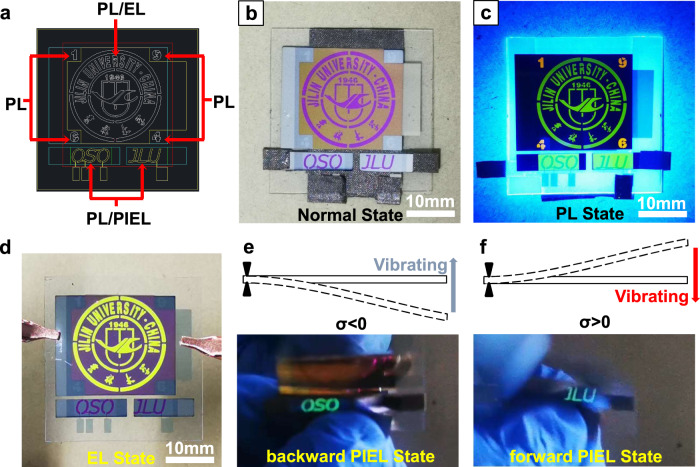
Different operation modes of the multi-functional anti-counterfeiting device. **a** Schematic diagram of the functional sections and structure of the demo pattern. Patterns of each color refer to different masks in OLED evaporation process. Optical images of different anti-counterfeiting modes are shown respectively as **b** normal state and **c** photoluminescence state (PL state), **d** electroluminescence state (EL state) and **e, f** Piezoelectrical-induced EL state (PIEL state) by free vibration with different initial deflection σ. Forward direction: σ > 0; Backward direction: σ < 0. The black triangles indicates the fixed end of the device.

Photoelectric anti-counterfeiting technology has received widespread attention. It is essentially a process of generating a signal output (luminescence) from one or more inputs (such as incident light, external force, electrical power supply, etc.) through physical principles encoding^60^. Through the method of multiple masks design and fabricating processes shown in Supplementary Fig. 7, a multi-functional anti-counterfeiting device capable of converting multiple inputs into patterned light output was realized. Figure 5a shows the functional partition and structure diagram of the anti-counterfeiting device. Specifically, the multi-functional anti-counterfeiting device is divided into three regions: the number 1946 as the PL region, the school badge pattern as the PL/EL region, and the words OSO and JLU as PL/PIEL region. Three regions share the same carrier transporting layer. The PL and PL/EL regions were patterned by emitter masks, while the PL/PIEL region was patterned by a unique cathode mask to adjust the driving current by area control while preventing the leakage current between cathode and carrier transporting layer. Figure 5b is a photo of the multi-functional anti-counterfeiting device in non-working state.

With no cathode coverage, the reflective cavity of PL region is missing, so the word 1946 is almost invisible under non-working state. When the PL pattern is irradiated with a violet lamp (365 nm), the phosphorescent dye bis (4-phenyl-thieno[3,2-c] pyridinato-N, C^2’^)(acetylacetonate) iridium(III) (PO-01) is excited to produce a bright orange PL emission. At the same time, other patterns are also changed from purple to the dye PL color (As shown in Fig. 5c). Interestingly, the presence of microcavity in the EL/PL region causes a blue shift in its PL spectrum, showing green emission. When the PL/EL region is driven by external DC power supply as shown in Fig. 5d, the school badge pattern presents a bright yellow EL emission. With the same phosphorescent dye PO-01, three different colors of light emission are obtained, thus further improing the security of the anti-counterfeiting device. In addition, different organic luminescent dyes can be applied to achieve diversified PL color output. The background of PL/EL region also shows a weak blue-violet luminescence due to the direct contact between the hole-transporting layer (HTL) and the electron-transporting layer (ETL) layer, generating a large leakage current and leading to EL between the layers.

Figure 5e, f shows the optical images of the patterned PIEL working states. Cu fiber tapes as conductive layers connect the positive and negative electrodes of the PG to the cathode and anode of the OSO respectively, and reversibly connect to the JLU pattern to realize bidirectional PIEL. A free vibration with an initial deflection in backward direction (here we define the TEOLED side of the flexible mechanoluminophore device as forward, while the PG side as backward) will lit up the OSO pattern. When the initial deflection is in forward direction, the reversed β-phase dipoles in the PVDF will generate an opposite current, thus lit up the JLU pattern. The Supplementary Movie 3 demonstrates the process of light pulse anti-counterfeiting signal generation. By integrating the three encoding processes of PL, EL, and PIEL, a multi-functional anti-counterfeiting device capable of converting mechanical, electrical, or optical input into pattern display is realized, which sheds light on drastically improving the security level of anti-counterfeiting technology.

To further illustrate the multi-stimuli activated organic mechanoluminophore device for Internet of Things (IoTs) applications, a quick response (QR) code system adopting ML, PL, and EL patterns, as shown in Supplementary Fig. 8, Supplementary Fig. 9, and Supplementary Note 4, is created. As shown in Supplementary Fig. 8, consumers can activate the ML, PL, and EL patterns that make up the QR code by finger movement, a cellphone LED, and a battery, respectively. After uploading photos of different patterns to the manufacturer’s server, the QR code will be reconstituted by computer and send to both consumers and manufacturer for commodity certificating. For manufacturers, the cost of expensive identification instruments can also be saved. Due to the UV excitation interference layer, the correct QR code image will not be displayed under UV excitation, which further improves the security authentication level. The conversion from physical signals to digital information is one such usage case that illustrates its IoT applicability.

## Discussion

In summary, we reported a low-cost flexible mechanoluminophore device based on a PEN substrate supported PVDF piezoelectric generator and high-efficiency, low-reflectance TEOLED. Under free vibrating *d*_31_ mode, the PG produces a current density as high as 0.64 mA cm^−2^ even when driving a 10 mm^2^ OLED. Through material selection, structure design, and microcavity modulation, we obtained stable electroluminescence with a turn-on voltage of ~3 V and turn-on current density <1 μA cm^−2^. By adding a rubrene capping layer, we improved the light-extraction efficiency of TEOLED and achieved luminous reflectance as low as 0.27. We applied equivalent intensity of light pulse and the Weber-Fechner’s law to mechanoluminophore devices, thus quantitatively characterizing the proposed flexible mechanoluminophore device with a physiological equivalent brightness of 11.5 cd m^−2^ and a just-noticeable-ambient-illuminance up to 3000 lux. The reported multi-functional anti-counterfeiting device combining electroluminescence, photoluminescence, and piezoelectric-induced mechanoluminophore suggests an avenue towards next-generation high-security level anti-counterfeiting technology and many others applications. The flexible mechanoluminophore device reported here can be integrated onto robotics, packages, toys, clothes, etc. towards various improved functions and utilities.

## Methods

### PG preparation

The double-sided aluminum-plated electrode PVDF film (50 μm, Kexin Electronics. Corp., China) was cut into 20 mm × 25 mm size, then covered with a double-sided conductive Cu fiber woven tape, and then made the PVDF aluminum electrode and Cu fiber tape in full contact. The film-coated PEN into a 20 mm × 25 mm base material was cut. After removing the protective film, the prepared Cu-PVDF-Cu sandwich structure laminated piezoelectric film was pasted on the PEN substrate, with the positive side of the PVDF facing the PEN substrate.

### OLED preparation

First, a 20 mm × 25 mm PEN film was cut, which acted as the device substrate. Then it was baked in an oven at 120 °C for 15 min to remove water molecules and volatile substances attached to the surface of the substrate. Then the protective film was removed, and a multi-source organic vapor deposition system (LN-182A, Lining Co., Ltd., China) was used to vaporize 100 nm aluminum electrode on it with a deposition rate of 1-2 Å/s. Then 3 nm MoO_3_ was deposited on it at a rate of 0.1-0.2 Å s^−1^, followed by deposition of *x*/5/20 nm thick di-[4-(N, N-di(p-tolyl)-amino)-phenyl] cyclohexane (TAPC)/4,4′,4′′-Tris(carbazol-9-yl) triphenylamine (TcTa) and a 4,4′-N, N′-dicarbazole (CBP):CzDBA layer with a host-guest doping ratio of 10:1 at the rate of 1-2, 0.5, and 1-2 Å s^−1^, respectively. The next step was to deposit a *y* nm thick 1,3,5-Tris(3-pyridyl-3-phenyl) benzene (TmPyPB) layer and a 0.5 nm LiF layer at the rate of 2 and 0.1 Å s^−1^, respectively. Finally, the Al/Ag thin film was deposited at a speed of 1-2 Å s^−1^ with a thickness of 20 nm. The thickness parameters, namely *x* and *y*, are adjustable in order to optimize the cavity length of TEOLED, ranging from 108.5 to 138.5 nm. Additionally, a rubrene capping layer was deposited on top of the TEOLED cathode. All the materials were purchased from Luminescence Technology Corp.

### Flexible mechanoluminophore device preparation

The TEOLED based on a PEN flexible substrate was prepared, and then a Cu-PVDF-Cu sandwich structure laminated piezoelectric film was pasted on the opposite side of the TEOLED. The PVDF positive and negative electrodes were connected with TEOLED cathode and anode, respectively, through branches of the conductive layers.

### Multi-functional anti-counterfeiting device preparation

As shown in Supplementary Fig. 7, several large-area patterned masks were used to prepare an electro-photo-piezoelectric-driven anti-counterfeiting pattern based on the PEN substrate with an area of 35 mm × 35 mm. The anode of PL/EL/PIEL sections was vacuum evaporated with 100 nm thick aluminum, followed by a HTL and electron blocking layer (EBL) of 40 nm TAPC and 5 nm TcTa. 20 nm CBP:PO-01 was deposited with a host-guest doping ratio of 20:1 as the PL&PL/EL emitter, while 20 nm CBP:CzDBA with a host-guest doping ratio of 10:1 as the PL/PIEL emitter. Then, a 60 nm TmPyPB and 0.5 nm LiF were deposited as ETL and electron injection layer (EIL). Finally, an Al (1 nm)/Ag (19 nm) thin film was evaporated as the cathode, where the PL section was uncovered in order to avoid microcavity effect, so that the PL section could only be seen under UV. Similarly, the PG with a size of 35 mm × 25 mm was prepared on the backside of the PEN to supply power for the flexible mechanoluminophore device.

### Characterization

The photoelectric performances of TEOLED were measured by a goniophotometric measurement system (GP500, Otsuka Electronics Co. Osaka, Japan). The piezoelectric electrical signal generated by PVDF and the optical signal generated by TEOLED were tested by an oscilloscope (DS4054, RIGOL, China), a fiber input Si biased detector (DET025AFC, Thorlabs, USA), a 3D printed test fixture, and a mechanical testing instrument (FT-8000D, Suzhou F-Tom testing equipment Co., Ltd, China).

## Supplementary information

Supplementary Information

Description of Additional Supplementary Files

Supplementary Movie 1

Supplementary Movie 2

Supplementary Movie 3

## Data Availability

The Source data that support the findings of this study are available in figshare with the identifier 10.6084/m9.figshare.22083347.v1^61^.
