# Supplementary material for: A flexible organic mechanoluminophore device
Source: Nat Commun. 2023 Mar 6;14:1257. doi: 10.1038/s41467-023-36916-z (PMC9988937; doi:10.1038/s41467-023-36916-z)
Supplement: Supplementary file 1 — Supplementary Information [file 41467_2023_36916_MOESM1_ESM.pdf]

Supplementary Information for

A flexible organic mechanoluminophore device

Qingyang Zhang[1], Mengxin Xu[1], Liming Zhou[2], Shihao Liu[1], Wei Wang[1], Letian Zhang[1], Wenfa Xie[1]*, and Cunjiang Yu[3,4,5]*

[1]State Key Laboratory of Integrated Optoelectronics, College of Electronic Science and Engineering, Jilin University, Changchun, 130012, China

[2]School of Mechanical and Aerospace Engineering, Jilin University, Changchun, 130025, China

[3]Department of Engineering Science and Mechanics, Pennsylvania State University, University Park, PA, 16802, USA

[4]Department of Biomedical Engineering, Pennsylvania State University, University Park, PA, 16802, USA

[5]Department of Material Science and Engineering, Materials Research Institute, Pennsylvania State University, University Park, PA, 16802, USA

Correspondence: Wenfa Xie (xiewf@jlu.edu.cn) or Cunjiang Yu (cmy5358@psu.edu)

Table of Contents:

I. Supplementary figures and tables

Supplementary Figure 1 Calculations and performances of the piezoelectric generator.

Supplementary Figure 2. Comparison between experimental and finite element analysis simulation results of electrical potential generated by damping PG.

Supplementary Figure 3. DC&AC properties of TEOLED.

Supplementary Figure 4. Influence of capping layer on optical reflection.

Supplementary Figure 5. Electrical performance of TEOLED devices.

Supplementary Figure 6. Cyclic bending test.

Supplementary Figure 7. The general process of fabricating multifunctional anti-counterfeiting devices by patterned mask and vacuum evaporation process.

Supplementary Figure 8. The realization process of quick response (QR) code system adopting ML, PL and EL patterns.

Supplementary Figure 9. The metal masks used in quick response (QR) code system adopting ML, PL and EL patterns.

Supplementary Table 1. Parameters for y_n calculation. h_i and y_i are the thickness and central plane-origin distance of each layer.

II. Supplementary notes

Supplementary Notes 1-4

III. Supplementary references

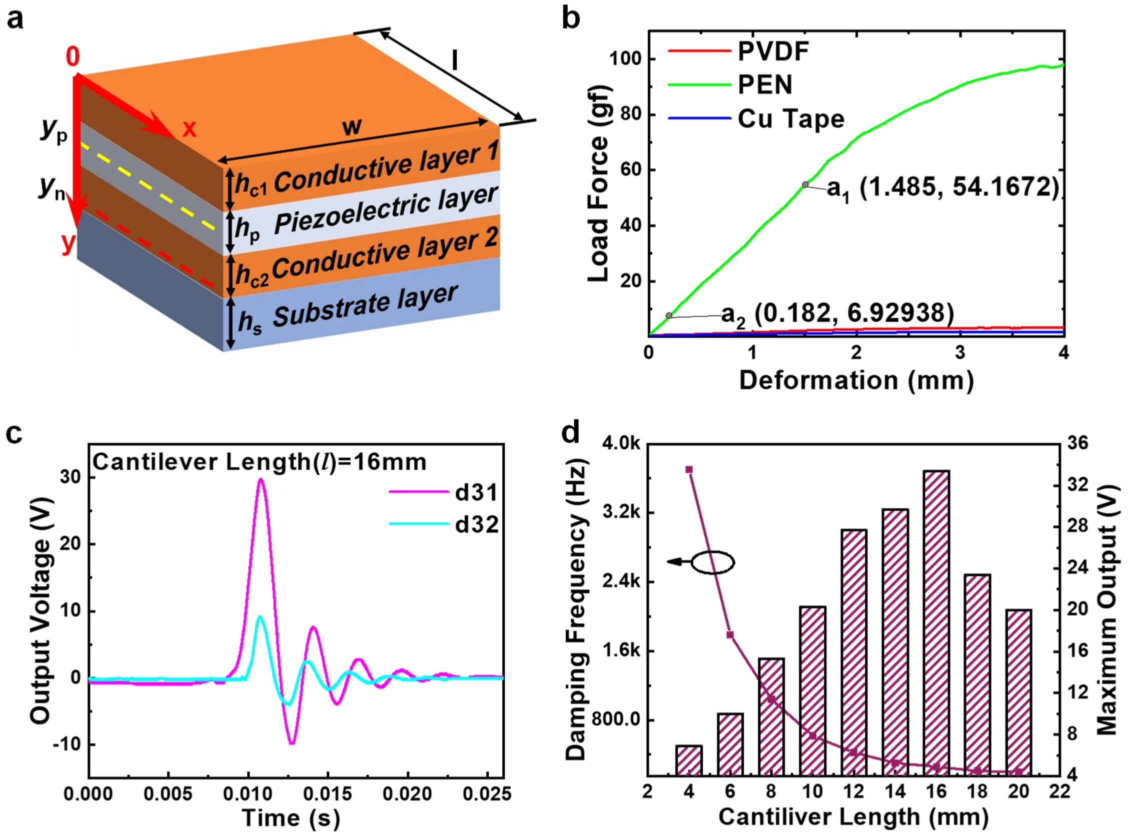

Supplementary Figure 1. Calculations and performances of the piezoelectric generator. a Coordinate settings of laminated cantilever beams. **b** Load-deformation curve of single layer material measured by three-point bending method. The material bending modulus of elasticity is calculated by taking two points a_1 and a_2 in the linear region of the force-displacement curve. **c** Output comparison under different piezoelectric film orientations. **d** PG peak voltage output (bar) and free vibration frequency (dot-line) as a function of the cantilever length.

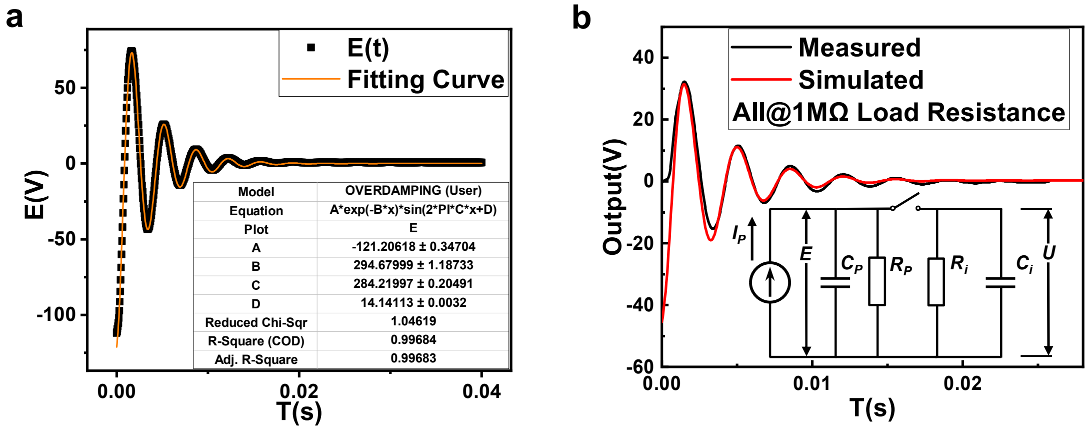

Supplementary Figure 2. Comparison between experimental and finite element analysis simulation results of electrical potential generated by damping PG. a Finite element simulation result of PG. The inset table shows the nonlinear fit parameters of the simulation. **b** Voltage output of a PG with 1MΩ load measured by oscilloscope and calculated from simulation result. The inset is an equivalent circuit for the whole construction.

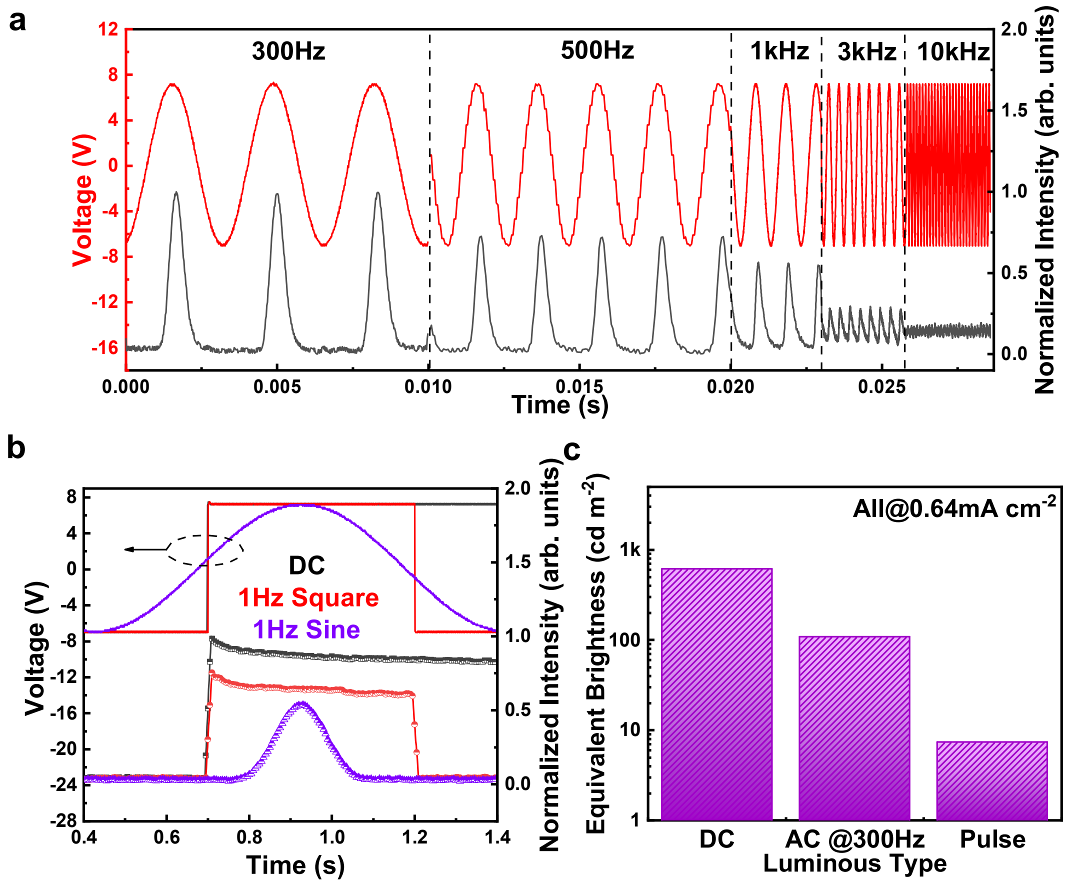

Supplementary Figure 3. DC&AC properties of TEOLED. a Dependence of driving voltage (red curve on top) and normalized EL intensity (black curve at bottom) on time at different frequencies. **b** Normalized EL Intensity of OLED (colored symbol-lines) under 7V@1Hz square wave/sine wave and DC power supply (colored solid lines). **c** Equivalent light intensity of TEOLED under different driving modes.

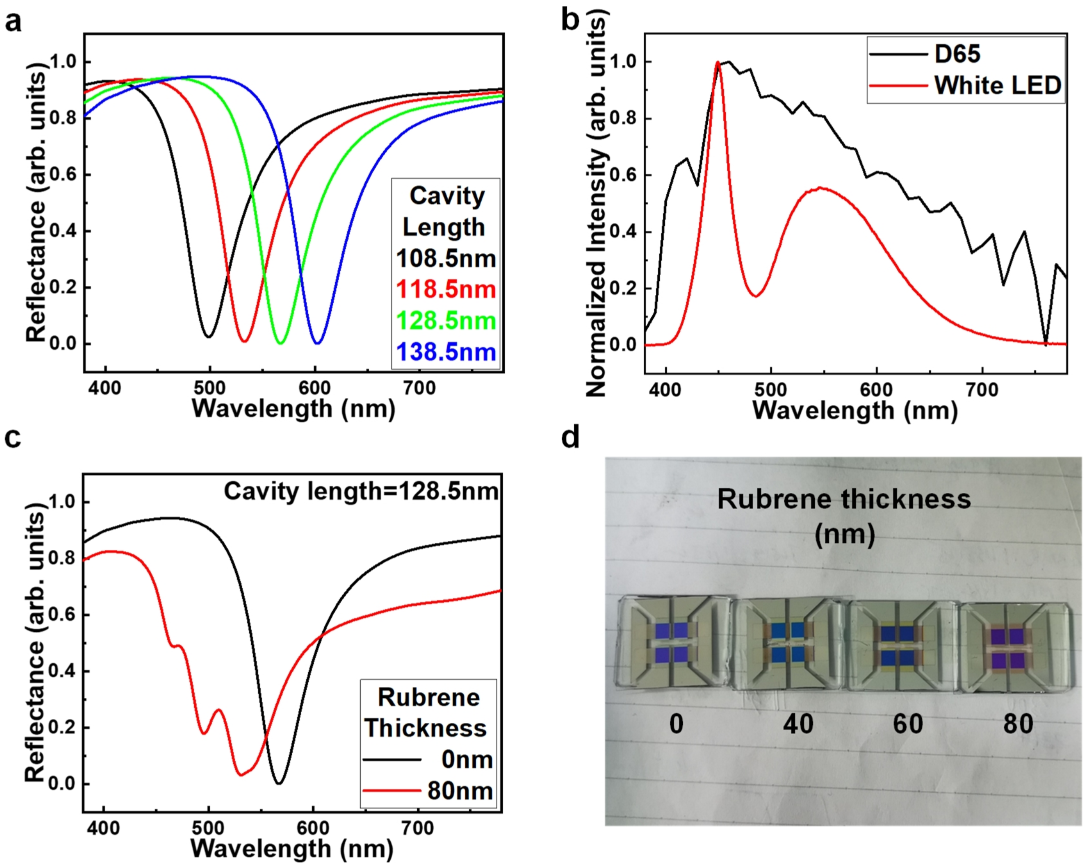

Supplementary Figure 4. Influence of capping layer on optical reflection. a Influence of cavity length on optical reflection. **b** EL spectra of white LED and D65(CIE) ambient light source. **c** Influence of rubrene capping layer on optical reflection. **d** Photos of high-contrast TELOEDs with varied rubrene thickness.

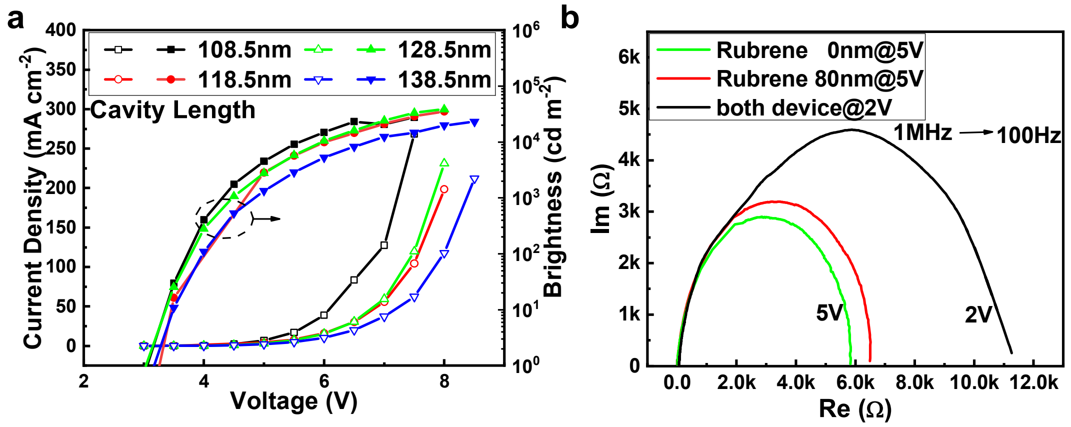

Supplementary Figure 5. Electrical performance of TEOLED devices. a I-V-B curves of TEOLEDs with different cavity lengths. **b** Cole-cole plot of TEOLED equivalent impedance.

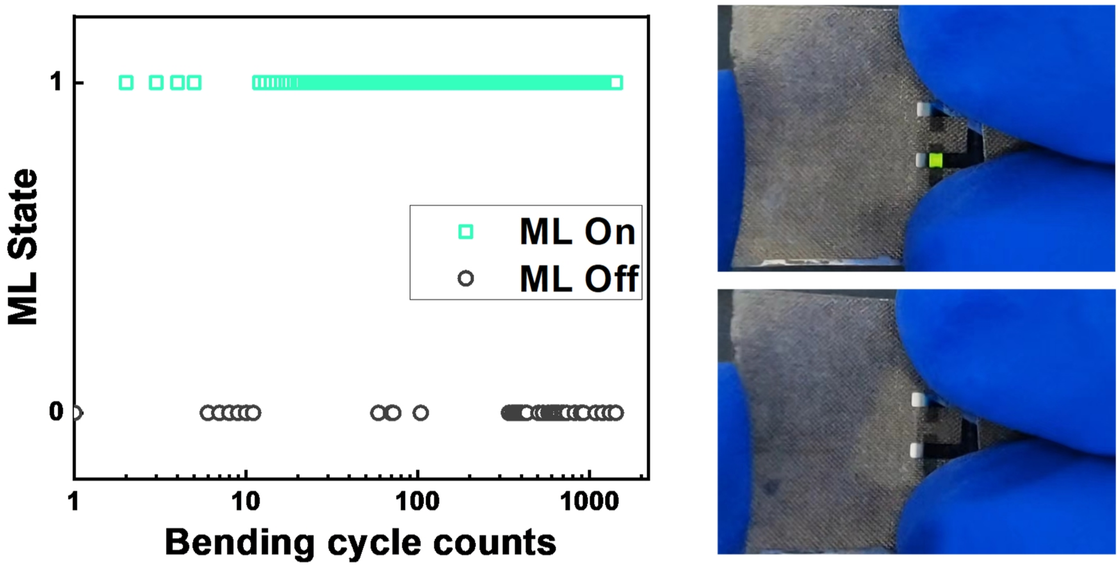

Supplementary Figure 6. Cyclic bending test. In the scheme, the ML state "1" in Y-axis refers to on state of OLED unit in a single bending behavior, vice versa. The photos on the right correspond to the ON/OFF states of the test device under a single external force input.

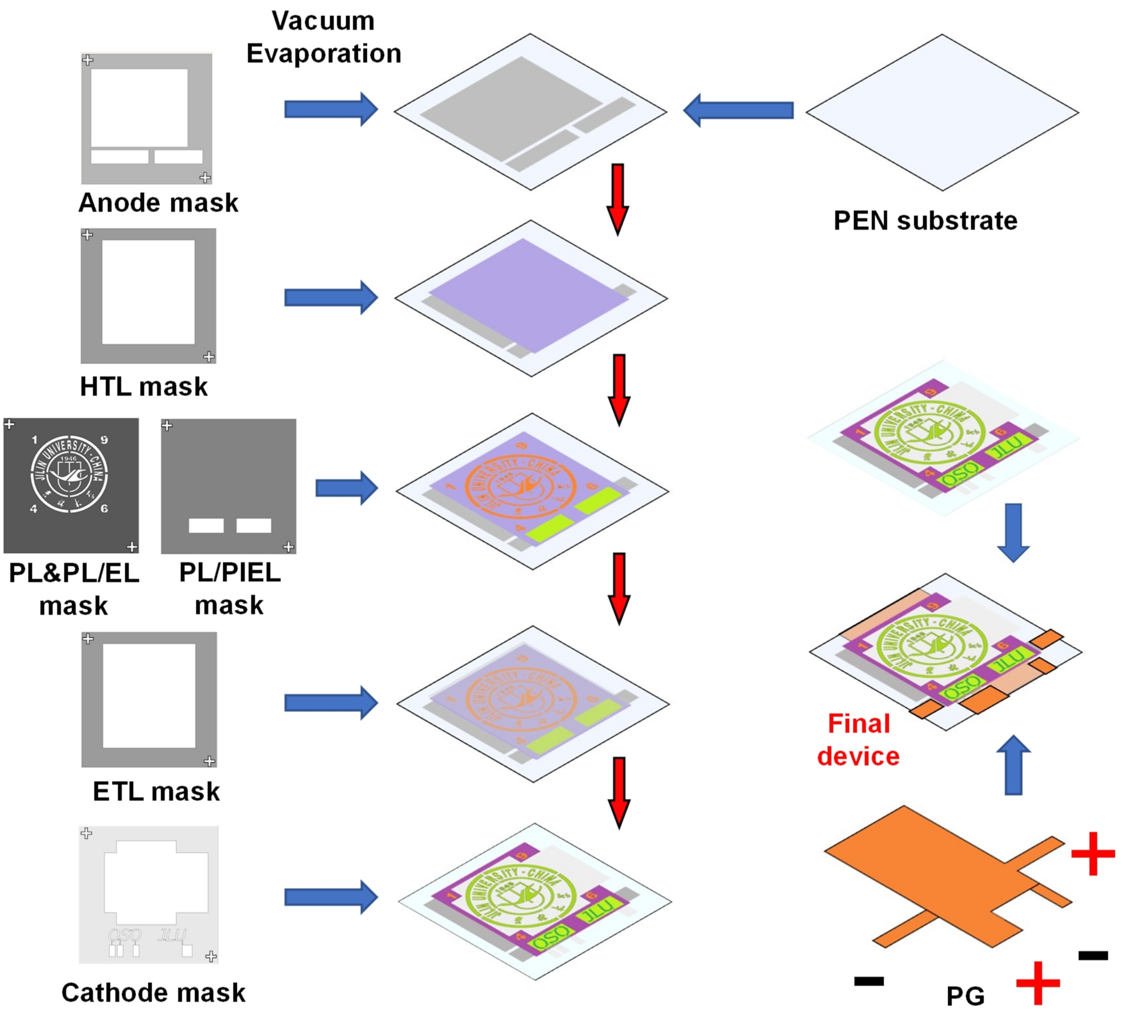

Supplementary Figure 7. The general process of fabricating multifunctional anti-counterfeiting devices by patterned mask and vacuum evaporation process. The left side of the picture shows the TEOLED evaporation process, and the lower right corner of the picture shows the assembly process of flexible mechanoluminophore device. The crosses at the upper left corner and lower right corner of each mask are designed for mask alignment.

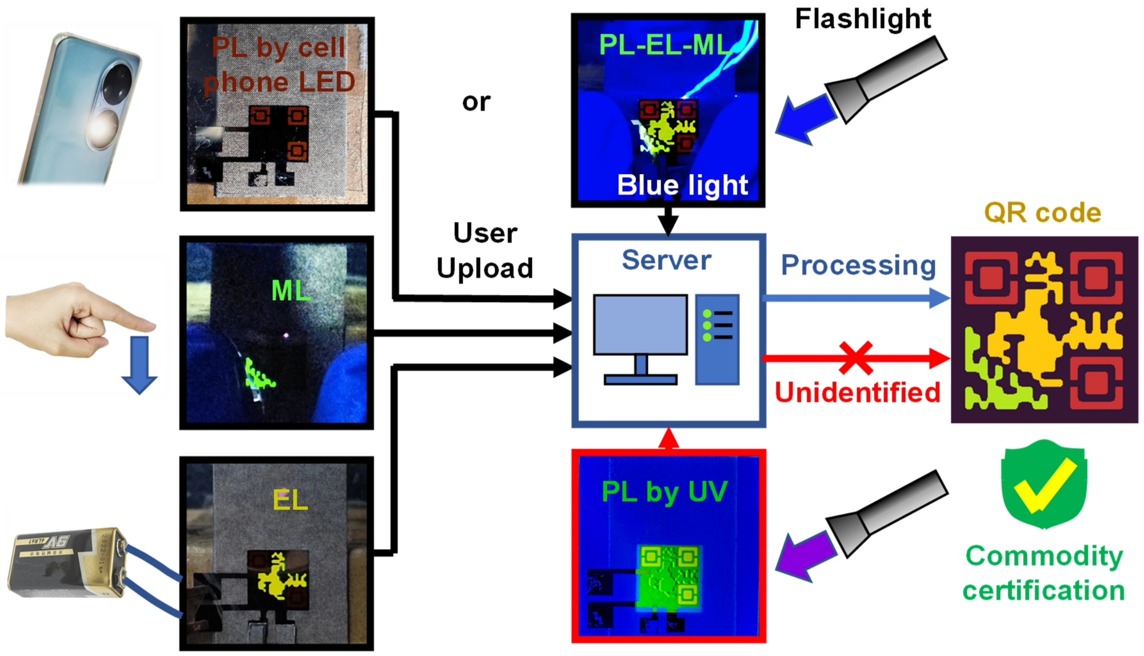

Supplementary Figure 8. The realization process of quick response (QR) code system adopting ML, PL and EL patterns. The corresponding optical signals are obtained through different external excitation, and are converted into effective anti-counterfeiting identification information through system processing. The application of flexible organic mechanoluminophore device has raised the technical barrier of imitation, thereby improving the safety. Two certification ways for consumers: (1)Activating three patterns simultaneously for direct scanning. (2)Acitvating three patterns respectively, uploading their photos to manufacturer's server and getting feedback after system processing.

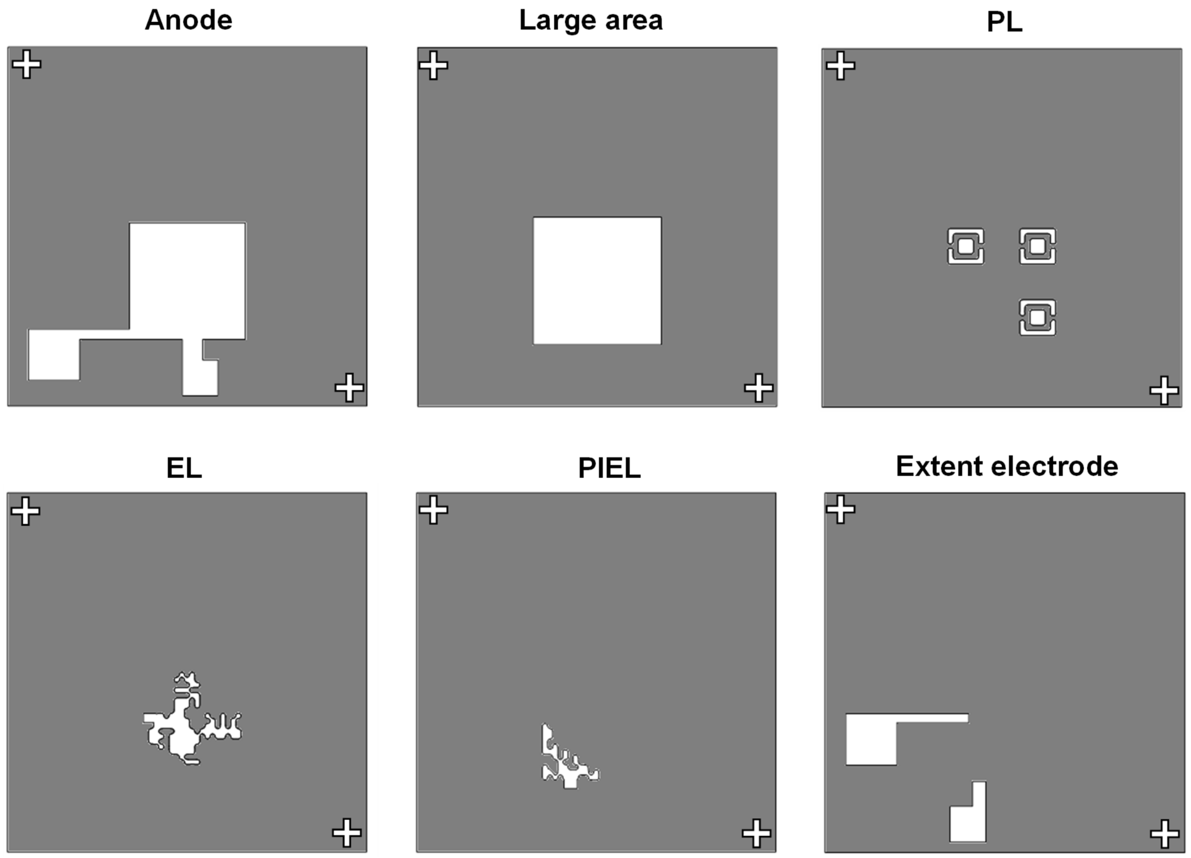

Supplementary Figure 9. The metal masks used in quick response (QR) code system adopting ML, PL and EL patterns. The crosses at the upper left corner and lower right corner of each mask are designed for mask alignment.

Supplementary Table 1. Parameters for y_n calculation. h_i and y_i are the thickness and central plane-origin distance of each layer.

Parameter \ Material	PEN	PVDF	Cu tape 1	Cu tape 2
E_i (MPa)	7528	3643	240	
h_i (μm)	125	50	125	
y_i (μm)	362.5	150	62.5	212.5

Supplementary Note 1:

As shown in Supplementary Fig. 1a, consider the top-left vertex of the conductive layer 1 as the origin 0, the direction along the length l of the cantilever as the x-axis, and the direction along the thickness direction as the y-axis. In the figure, h_i is the thickness of the corresponding layer, and w is the width of the cantilever beam. The distance from the origin 0 to the center planes (shown by the yellow dotted lines) of the piezoelectric layer,the conductive layers and substrate layer are y_p, y_c and y_s,respectively. The distance from the origin 0 to the neutral surface of the laminated beam (red dotted line) is y_n.

Before calculating the distance y_n between the median plane and the origin, we first determine and calculate the bending elastic modulus E_i of each layer of material. The experiment uses a three-point measurement method and uses a single-layer material with the same length and width of laminated cantilever beams for testing. The test results are shown in Supplementary Fig. 1b. Take the PEN layer as an example, pick two points a_1 and a_2 on the curve that are far apart in the linear zone, and calculate the bending elastic modulus E_i by the following equation:

$$E_i = \frac{l_0^3}{4wh^3} \times \frac{\Delta P}{\Delta y} = \frac{l^3(P_1-P_2)}{4wh^3(y_1-y_2)} \qquad (1)$$

Where l_0 is the span of the test bracket, P is the load force, and y is the deformation. Substituting a_1 and a_2 into the above equation, E_{PEN}=7528 MPa can be obtained. In the same way, E_{PVDF}=3643 MPa and E_{Cu}=240 MPa can also be obtained (Cu tape is a flexible braided layer, so the bending strength is low). The required parameters in the main text Equation (1) are listed in Supplementsry Table 1, for two layers of Cu tape, the equation should be given by[1]:

$$y_{\mathrm{n}} = \frac{E_{\mathrm{p}} y_{\mathrm{p}} h_{\mathrm{p}} + E_{\mathrm{s}} y_{\mathrm{s}} h_{\mathrm{s}} + E_{\mathrm{c}} y_{\mathrm{c}} (h_{\mathrm{c}_1} + h_{\mathrm{c}_2})}{E_{\mathrm{p}} h_{\mathrm{p}} + E_{\mathrm{s}} h_{\mathrm{s}} + 2 E_{\mathrm{c}} h_{\mathrm{c}}} \quad (2)$$

Substituting the parameters into the equation and get y_{n}=319 μm.

To characterize the piezoelectric output of the cantilever, one short side of the cantilever is fixed, and the opposite short side is left as free end. An initial deflection is applied and released at the free end to create free vibration. The relationship between the cantilever length *l* of the Laminated beam, the deflection $\delta(x, t)$ at time *t,* and arm length *x* during free vibration and the voltage output is given by[2]:

$$V(t) = \frac{Q}{C} = \frac{h_p d_{31}}{\varepsilon l s_{11}} (y_{\mathrm{n}} h_{\mathrm{p}} - \frac{1}{2} h_{\mathrm{p}}{}^2)(\frac{\partial^3 \delta(l,t)}{\partial x^3} - \frac{\partial^3 \delta(0,t)}{\partial x^3}) \quad (3)$$

where ε and s_{11} are the dielectric constant and elastic compliance coefficient of PVDF, respectively. It can be seen that the farther the neutral plane is from the center of the piezoelectric layer, the greater the voltage output, which theoretically proves the necessity of adding a PEN support layer.

Supplementary Note 2:

We used ABAQUS software for carrying out finite element simulation of PG voltage output. The cantilever length is set to 16 mm, the initial displacement of the free end is 10 mm, and a 1 N plane force is applied on the 9 mm range of the free end to simulate the downward pressure of fingers. In order to simplify the calculation, without considering the tangential strain and contact loss, the voltage output curve as shown in Supplementary Fig. 2a is obtained. In order to calculate the output voltage connected to the load circuit, we have performed nonlinear fitting on the output voltage *E*(*t*). The fitting curve and parameter table are shown in the inset of Supplementary Fig. 2a. The equivalent circuit diagram with resistive load and parasitic capacitance is shown in the Supplementary Fig. 2b. For the convenience of calculation, the contact resistance has been ignored. When the circuit is open, the potential difference between the two plates is E, then the current I_{P} of the controlled current source is calculated as follows:

$$I_{\mathrm{P}} = \frac{dQ}{dt} + \frac{E}{R_{\mathrm{P}}} = C_P\frac{dE}{dt} + \frac{E}{R_{\mathrm{P}}} \tag{4}$$

Where $R_{\mathrm{P}} = 10^{12}\Omega$ is the internal resistance of the PG and C_{P}=0.753 nF is the capacitance between two electrodes. After connecting the load $R_{\mathrm{i}} = 1\ \mathrm{M\Omega}$ and the parasitic capacitance $C_{\mathrm{i}} = 1.1\ \mathrm{nF}$ in parallel, according to Kirchhoff's current theorem:

$$(C_{\mathrm{P}} + C_{\mathrm{i}})\frac{dU}{dt} + \frac{U}{R_{\mathrm{i}}} + \frac{U}{R_{\mathrm{P}}} = I_{\mathrm{P}} \tag{5}$$

Combining Supplementary Equation (4) and Supplementary Equation (5) and performing Laplace transform, we get:

$$U(\boldsymbol{s}) = \frac{R_{\mathrm{i}}+R_{\mathrm{i}}R_{\mathrm{P}}C_{\mathrm{P}}s}{[s(C_{\mathrm{P}}+C_{\mathrm{i}})+1](R_{\mathrm{i}}+R_{\mathrm{P}})}E(\boldsymbol{s}) \tag{6}$$

Taking the inverse Laplace transform of the above formula, we get:

$$\mathcal{L}^{-1}[U(\boldsymbol{s})] = U(t) = \frac{R_{\mathrm{i}}R_{\mathrm{P}}C_{\mathrm{P}}}{(C_{\mathrm{P}}+C_{\mathrm{i}})(R_{\mathrm{i}}+R_{\mathrm{P}})}E(t) + \frac{R_{\mathrm{i}}(C_{\mathrm{P}}+C_{\mathrm{i}}-R_{\mathrm{P}}C_{\mathrm{P}})}{(C_{\mathrm{P}}+C_{\mathrm{i}})^2(R_{\mathrm{i}}+R_{\mathrm{P}})}\int_0^t e^{\frac{-u}{(C_{\mathrm{P}}+C_{\mathrm{i}})}}E(t-u)du \tag{7}$$

The parameters of each circuit element and the open-circuit voltage waveform $E(t)$ obtained by finite element analysis are brought into the above formula, and the voltage $U(t)$ on the load can be obtained, as shown in Supplementary Fig. 2b.

Supplementary note 3:

In order to quantitatively describe the ambient light tolerance of the integrated device through Fechtner-Weber's law, we first need to measure the pulsed light equivalent intensity of the OLED. Considering the use of the small-resistor series method to determine the OLED drive current, the impedance spectroscopy of the OLED is measured. As shown in Supplementary Fig. 5, the OLED can be considered as a pure resistance at low frequency, and the working impedance is about 6500 Ω. Therefore, a small resistance R_0=100 Ω was connected in series with an OLED, and the voltage across R_0 was measured to obtain the OLED operating current J_{OLED}=V_{R0}/R_0. As shown in Fig. 4d, the corresponding DC light intensity I_{0DC} is found in the DC electrical characteristics of the OLED through the current (J_{OLED}) amplitude of 0.64 mA cm^{-2}, and the intensity amplitude I_0 of the light pulse is calculated according to the following equation:

$$I_0 = AI_{0\mathrm{DC}} \tag{8}$$

where A=0.875 is the AC/DC attenuation factor at the PG driving frequency, which represents the ratio of AC peak light intensity to DC light intensity under the same driving voltage/current.

Supplementary note 4:

By adopting the method of sub-section mask evaporation, which is similar to the multi-function anti-counterfeit devices in the manuscript, we have prepared a multi-stimuli activated QR code system that can be used for commodity verification.

Preparation process: All masks used in the preparation process are shown in Supplementary Fig. 9. Firstly, a 100 nm thick Al film was deposited as anode (anode mask) on a 35 mm×35 mm PEN substrate. A hole injection layer/HTL/EBL layers of MoO_3 (3 nm) /TAPC (40 nm)/TcTa (5 nm) were deposited using the large area mask. It is noted that the overlay alignment was complished based on the cross shaped alignment markers at the upper left and lower right conerns of each mask. Next, a 15 nm thick C545T: DCJTB (50:1) layer was deposited as PL section through the PL mask. A 20 nm thick CBP: PO-01 (20:1) and 20 nm thick CBP: CzDBA (10:1) layers were deposited as the EL section and the ML section, respectively, by using EL and PL masks. Then a 60 nm thick TmPyPB and a 0.5 nm thick LiF were deposited as the ETL and EIL through the large area mask. The cathode consisting of 1 nm Al/19 nm Ag for EL and ML sections was deposited separately through the EL and ML masks. For easier electrical connection to power source, extent electrodes of 50 nm thick Al were deposited on the device. After that, a 120 nm CBP:CzDBA was deposited as UV excitation interference layer through the large area mask. Finally, a 35 mm×25 mm PG was fabricated and assembled to the back of the PEN substrate, with its electrodes connecting to the ML section.

Supplementary references

1. Kim, D. H. et al. Stretchable and foldable silicon integrated circuits. *Science* **320**, 507-511 (2008).

2. Hong, Y. et al. Theoretical analysis and experimental study of the effect of the neutral plane of a composite piezoelectric cantilever. *Energy Convers. Manage.* **171**, 1020-1029 (2018).
