# Supplementary material for: A flexible organic mechanoluminophore device
Source: Nat Commun. 2023 Mar 6;14:1257. doi: 10.1038/s41467-023-36916-z (PMC9988937; doi:10.1038/s41467-023-36916-z)
Supplement: Supplementary file 2 — Description of Additional Supplementary Files [file 41467_2023_36916_MOESM2_ESM.pdf]

Description of Additional Supplementary Files

File name: Supplementary Movie 1

Description: Piezoelectric generator lights up TEOLED. When the optimized TEOLED is connected to the piezoelectric generator by wires, green light pulses can be successfully observed by actuating the PG.

File name: Supplementary Movie 2

Description: Duration test of the flexible organic mechanoluminophore device. The device was bent over 1300 times under ambient environment.

File name: Supplementary Movie 3

Description: The process of light pulse anti-counterfeiting signal generation. By bending and vibrating in opposite directions, the device generates different EL patterns.
